# Extraintestinal *Entamoeba moshkovskii* Infection, Eastern India

**DOI:** 10.3201/eid3203.251065

**Published:** 2026-03

**Authors:** Sanjib Kumar Sardar, Ajanta Ghosal, Tapas Haldar, Basudev Ganguly, Koushik Das, Sandipan Ganguly

**Affiliations:** The University of Tokyo, Bunkyo, Japan (S.K. Sardar); ICMR–National Institute for Research in Bacterial Infections, Kolkata, India (S.K. Sardar, A. Ghosal, T. Haldar, B. Ganguly, S. Ganguly); Assam University, Guwahati, India (K. Das).

**Keywords:** *Entamoeba moshkovskii*, parasites, pathogen, splenic abscess, extraintestinal infection, India

## Abstract

*Entamoeba moshkovskii* is historically considered nonpathogenic. We report a case of severe extraintestinal infection in a patient from eastern India who had abdominal pain, fever, weight loss, anemia, and a splenic abscess. Molecular analysis confirmed *E. moshkovskii* as the causative agent. This case highlights this parasite’s potential to cause severe illness.

For more than a century, the *Entamoeba histolytica* protozoan was considered the sole pathogenic amoeba to cause diarrhea, dysentery, and liver abscess. Microscopy was once the diagnostic standard, but because of the morphologic similarity among *Entamoeba* species, PCR is now recommended (*1*,*2*). Major morphologically identical species indistinguishable from *E. histolytica* include *E. dispar*, *E. moshkovskii*, and *E. bangladeshi* (*3*,*4*). This group of organisms (with *E. nuttalli*) is called the *E. histolytica* complex. Within this group, *E. moshkovskii* was historically regarded as nonpathogenic; however, emerging evidence suggests potential pathogenicity (*5*–*9*). Reported prevalence of *E. moshkovskii* varies widely (≈1%–25%) across epidemiologic settings (*5*–*10*). 

In Bangladesh, one study reported a 21.1% infection rate in children, whereas another reported a 2.95% prevalence with an association to diarrhea in infants (*8*,*11*). Similarly, a 3-year surveillance study in eastern India identified a 3.12% prevalence among diarrheal patients, supporting its classification as an emerging pathogen rather than a nonpathogenic species (*5*,*6*). In a murine model of intestinal amebiasis, *E. moshkovskii* was also found to cause diarrhea, weight loss, and colitis (*8*).

Emerging epidemiologic evidence suggests *E. moshkovskii* is a potential enteropathogen. We present a clinically documented case of an extraintestinal infection caused by *E. moshkovskii* that resulted in severe health complications in a patient from eastern India.

## The Study

A 36-year-old man from Kolkata, in West Bengal, India, sought care in July 2024 for a 6-day history of intermittent fever, characterized by 1–2 spikes per day and a recorded high temperature of 102°F. The patient reported fever with malaise but no chills or rigors, along with a 5-day history of dull, continuous left upper quadrant abdominal pain that worsened with deep inspiration, sneezing, or when lying in the left lateral decubitus position. A slow-developing heavy feeling in the left upper abdomen, along with nausea, a bad taste in the mouth, and 1 episode of nonbilious vomiting, also occurred. The patient reported weight loss from 53 kg to 47 kg over 4 weeks, despite preserved appetite. In addition, he had a history of occasional nonproductive cough without wheezing, hemoptysis, or shortness of breath. The patient had a left-lobe hepatic abscess diagnosed in March 2023 in Kolkata and was treated empirically with intravenous injection and oral metronidazole. The causative agent was not identified. The bad taste in the patient’s mouth was likely not related to the previous metronidazole treatment, because he had not been on the medication in the previous 3 weeks. 

The patient was a cigarette smoker but had abstained from alcohol for 4 years. On examination, his heart rate was 104 beats/min and blood pressure 146/74 mmHg; oxygen saturation (98% room air) and respiratory rate (16 breaths/min) were unremarkable. Physical examination revealed pallor, hepatomegaly (2 cm below right costal margin), splenomegaly (6 cm below left costal margin), and left upper quadrant tenderness. The patient had absent breath sounds over the left intercostal and subaxillary areas but showed no signs of cyanosis, clubbing, or lymphadenopathy. Neurologic examination results were unremarkable. Iron deficiency anemia was noted. Serologic testing revealed negative results for HIV, hepatitis B surface antigen, hepatitis C virus antibody, malarial parasite detection antigen, and *Brucella* IgM and IgG. The patient also had a nonreactive Rose Bengal plate test and standard tube agglutination test for *Brucella*. A chest radiograph revealed an elevated left hemidiaphragm and left pleural effusion. An abdominal ultrasound revealed hepatomegaly and a large splenic abscess (18 cm x 14 cm × 17 cm, 2000 mL) with mild subdiaphragmatic fluid. Contrast-enhanced computed tomography of the chest and abdomen confirmed a collapsed left lower lobe and a splenic abscess. The patient was managed with drainage and supportive care. 

**Figure 1:**
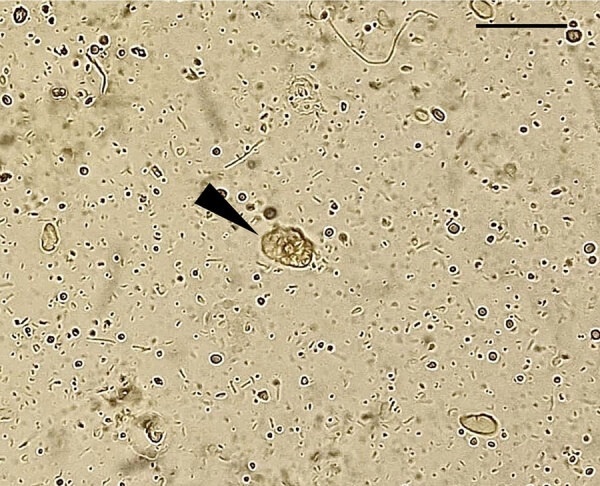
Microscopic view of *Entamoeba moshkovskii* trophozoites (arrowhead) observed in a pleural fluid sample recovered from a patient with an extraintestinal infection, eastern India. Slide used a wet-mount preparation,. Scale bar indicates 30 μm.

Pleural fluid analysis with Gram and Ziehl-Neelsen stains showed no microorganisms, and *Mycobacterium tuberculosis* was not detected. The wet mount showed predominantly macrophages, erythrocytes, epithelial cells, and many protozoal structures resembling *E. histolytica* with erythrophagia (Figure 1). However, splenic abscess aspirate did not reveal amoebae or amoeba-like structures. The direct smear revealed degenerated blood elements and necrosis, with no granuloma or malignant cells. We conducted additional characterization of the *E. histolytica*–like organism by using molecular methods.

**Figure 2:**
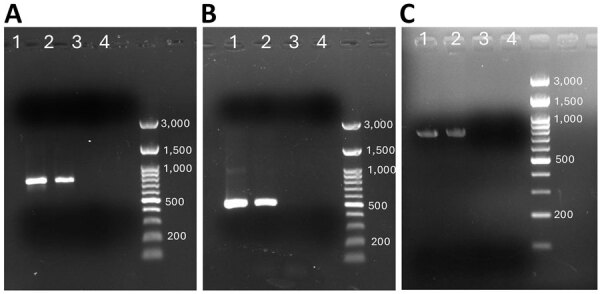
PCR amplification of DNA from an extraintestinal *Entamoeba moshkovskii* infection in a patient from eastern India. A) PCR amplification of the 18S rRNA locus of *E. moshkovskii* using DNA extracted from a splenic aspirate. The expected product size is 779 bp. B) PCR amplification of the chitinase locus of *E. moshkovskii* using DNA extracted from spleen aspirate samples. The expected product size is 480 bp. C) Amplification of the 18S rRNA locus of *E. moshkovskii* by PCR using DNA extracted from a stool sample. The expected product size is 779 bp. Lane 1, PCR product from patient sample; lane 2, positive control (*E. moshkovskii* genomic DNA); lane 3, *E. histolytica* genomic DNA; lane 4, negative control.

We extracted DNA by using the QIAamp DNA Mini Kit (QIAGEN, https://www.qiagen.com). We performed conventional PCR targeting the 18S rRNA gene on splenic aspirate and pleural fluid by using previously published species-specific primers for *E. histolytica* and *E. moshkovskii* (*5*,*12*). Both the splenic abscess aspirate and pleural fluid DNA tested negative for *E. histolytica* but positive for *E. moshkovskii* (Figure 2, panel A). To confirm the presence of *E. moshkovskii*, we targeted the chitinase gene for testing. We designed primer sequences to amplify the upstream and downstream regions of the chitinase open reading frame (Appendix Table). Both splenic abscess aspirate and pleural fluid samples again tested positive for *E. moshkovskii* when targeting the chitinase locus (Figure 2 panel B). We then purified the PCR products by using the Roche PCR Gel Extraction kit (Roche, https://www.roche.com) and sequenced bidirectionally with the BigDye Terminator v3.1 kit (Thermo Fisher Scientific, https://www.thermofisher.com). 

**Figure 3:**
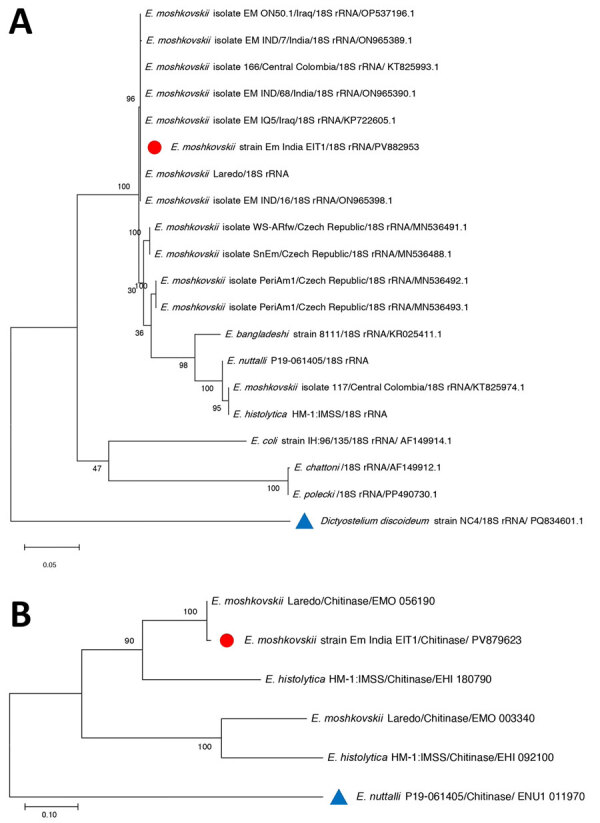
Phylogenetic trees constructed from the 18S rRNA and chitinase gene sequences obtained from *Entamoeba moshkovskii* isolates from a patient in eastern India (red circles) and reference sequences. A) Phylogenetic tree of 18S rRNA. The tree with the highest log-likelihood value (–2,562.32) is shown. Initial heuristic trees were generated automatically using the maximum parsimony approach. The model incorporated a proportion of invariant sites (45.63%). The analysis included 9 nucleotide sequences with 859 aligned positions in the final dataset. B) Phylogenetic tree of chitinase gene sequence. The tree with the highest log-likelihood value (−3,301.96) is presented. Initial heuristic searches were performed automatically using the maximum parsimony method. The final analysis included 20 nucleotide sequences, with a total of 939 positions in the final dataset. GenBank accession numbers are provided. Outgroup taxa are indicated with blue triangles. The phylogeny was inferred using the maximum likelihood method. Scale bars indicate substitutions per site.

BLAST analysis (https://blast.ncbi.nlm.nih.gov) confirmed the organism as *E. moshkovskii*, showing 100% identity to the reference Laredo strain (18S rRNA; GenBank accession no. AF149906.1). However, the chitinase gene corresponding to AmoebaDB (https://amoebadb.org/amoeba/app) entry EMO_056190 exhibited 3 mutations at positions 36 T/C, 127 G/A, and *2 T/G. The mutation at position 36 was synonymous, whereas the substitution at position 127 resulted in a nonsynonymous change, replacing glutamic acid with lysine. We constructed phylogenetic trees generated from the obtained 18S rRNA and chitinase gene sequences to confirm species identity and evaluate evolutionary relationships (Figure 3, panels A, B).

Of note, PCR analysis of DNA isolated from the patient’s stool samples by using the 18S rRNA targeted assay detected the presence of *E. moshkovskii* and not *E. histolytica* (Figure 2, panel C). In addition, results of ELISA testing for *E. histolytica*–specific IgG in serum were negative. Taken together, those findings indicate that neither the hepatic abscess nor the splenic abscess was associated with *E. histolytica* infection.

The patient was initially started on intravenous (IV) meropenem empirically. However, after confirmation of *E. moshkovskii* infection, we changed the treatment to IV metronidazole (750 mg IV/8 h for 1 d), followed by oral metronidazole (400 mg 3×/d after food for 7 d). The patient was also prescribed ferrous sulfate 200 mg (1 dose by mouth), B-complex tablets, and other medications as needed. Pigtail drainage continued for the first week. The patient recovered after 3 weeks.

## Conclusions

*E. moshkovskii* DNA alongside *E. histolytica* in 5 of 115 liver abscess cases was previously reported; however, extraintestinal *E. moshkovskii* infection, particularly in pleural fluid, is extremely rare (*13*). In the case we report, *E. moshkovskii* might have reached the pleural cavity through direct extension or rupture of a splenic abscess.

Metronidazole is the primary drug for treating amoebiasis and was effective in this patient, showing empirical activity against *E. moshkovskii* (*14*). However, the optimal dosage for this species remains undefined. Drug sensitivity differs among *Entamoeba* spp.; for instance, *E. moshkovskii* is resistant to emetine, which is effective against *E. histolytica* (*15*). Diagnosis is also challenging. Molecular tests often target only *E. histolytica*, leaving *E. moshkovskii* infections unrecognized. In addition, *E. moshkovskii* produces fewer cysts than *E. histolytica*, reducing the reliability of microscopic detection and increasing the risk for false negatives (*5*).

Because of the high regional prevalence of *E. moshkovskii*, extraintestinal infections caused by this species need attention. Our case underscores the need to better understand transmission and to develop improved diagnostic methods to ensure effective management and prevent drug resistance in *E. moshkovskii*.

Appendix Additional information about extraintestinal *Entamoeba moshkovskii* infection, eastern India.
