# Supplementary material for: Extraintestinal Entamoeba moshkovskii Infection, Eastern India
Source: Emerg Infect Dis. 2026 Mar;32(3):428–32. doi: 10.3201/eid3203.251065 (PMC13016018; doi:10.3201/eid3203.251065)
Supplement: Appendix — Additional information about extraintestinal Entamoeba moshkovskii infection, eastern India. [file 25-1065-Techapp-s1.pdf]

Article DOI: https://doi.org/10.3201/eid3203.251065

EID cannot ensure accessibility for supplementary materials supplied by authors. Readers who have difficulty accessing supplementary content should contact the authors for assistance.

Extraintestinal *Entamoeba moshkovskii* Infection, Eastern India

Appendix

Appendix Table. List of primers used in this study. Primers adopted from published sources were used with the reported annealing temperatures, and the original protocols were followed unless specified otherwise. The chitinase primer set was optimized in-house and confirmed to show no cross-amplification with closely related species, including *E. histolytica* and *E. dispar*. PCR amplification was performed using Takara Ex-Taq, following the manufacturer's recommended protocol.

Primer Name	Sequence (5′→3′)	Target gene/region	Amplicon Size (bp)	Annealing Temp (°C)	Tm (°C)	Reference
EH1	GTACAAAATGGCCAATTCATTCAATG	18S rRNA	135 bp	50°C	54 °C	(*1*)
EH2	ACTACCAACTGATTGATAGATCAG	18S rRNA			52 °C	(*1*)
EM_779bpNF	AACTAACGAAGGAGATGAAGTGAG	18S rRNA	779 bp	52 °C	55 °C	(*2*)
EM_779bpNR	GCCAGAGACATCGATTAAAATG	18S rRNA			52 °C	(*2*)
EmChitinase_F	TGTTGTTTGGAGAAATGAAAAGG	Chitinase gene upstream region	480 bp	50 °C	51 °C	This study
EmChitinase_R	TTGATGTTGCCCTCACTGAC	Chitinase gene downstream region			52 °C	This study

References

1. Mukherjee AK, Chowdhury P, Bhattacharya MK, Ghosh M, Rajendran K, Ganguly S. Hospital-based surveillance of enteric parasites in Kolkata. BMC Res Notes. 2009;2:110. PubMed https://doi.org/10.1186/1756-0500-2-110

2. Sardar SK, Ghosal A, Haldar T, Maruf M, Das K, Saito-Nakano Y, et al. Prevalence and molecular characterization of *Entamoeba moshkovskii* in diarrheal patients from Eastern India. PLoS Negl Trop Dis. 2023;17:e0011287. PubMed https://doi.org/10.1371/journal.pntd.0011287
